# Conversion of polyploid and alloploid *Saccharomyces* sensu stricto strains to *leu2* mutants by genome DNA editing

**DOI:** 10.1007/s00253-024-13242-y

**Published:** 2024-07-12

**Authors:** Kazuya Kiyokawa, Tetsushi Sakuma, Kazuki Moriguchi, Minetaka Sugiyama, Takeshi Akao, Takashi Yamamoto, Katsunori Suzuki

**Affiliations:** 1 Program of Basic Biology, Graduate School of Integrated Sciences for Life, Hiroshima University, Higashi-Hiroshima, Hiroshima, 739-8526 Japan; 2 Program of Mathematical and Life Sciences and Frontier Development Program for Genome Editing, Graduate School of Integrated Sciences for Life, Hiroshima University, Higashi-Hiroshima, Hiroshima, 739-8526 Japan; 3 Department of Food Sciences and Biotechnology, Faculty of Life Sciences, Hiroshima Institute of Technology, Hiroshima City, Hiroshima, 731-5193 Japan; 4 National Research Institute of Brewing, Higashi-Hiroshima City, Hiroshima, 739-0046 Japan; 5 Genome Editing Innovation Center, Hiroshima University, Higashi-Hiroshima, Hiroshima, 731-5193 Japan

**Keywords:** Genome editing, Guide RNA, Industrial yeast, Yeast isolates from natural sources, Prototroph, Template DNA

## Abstract

**Abstract:**

A large number of recombinant plasmids for the yeast *Saccharomyces cerevisiae* have been constructed and accumulated over the past four decades. It is desirable to apply the recombinant plasmid resources to *Saccharomyces* sensu stricto species group, which contains an increasing number of natural isolate and industrial strains. The application to the group encounters a difficulty. Natural isolates and industrial strains are exclusively prototrophic and polyploid, whereas direct application of most conventional plasmid resources imposes a prerequisite in host yeast strains of an auxotrophic mutation (i.e., *leu2*) that is rescued by a selection gene (e.g., *LEU2*) on the recombinant plasmids. To solve the difficulty, we aimed to generate *leu2* mutants from yeast strains belonging to the yeast *Saccharomyces* sensu stricto species group by DNA editing. First, we modified an all-in-one type CRISPR-Cas9 plasmid pML104 by adding an antibiotic-resistance gene and designing guide sequences to target the *LEU2* gene and to enable wide application in this yeast group. Then, the resulting CRISPR-Cas9 plasmids were exploited to seven strains belonging to five species of the group, including natural isolate, industrial, and allopolyploid strains. Colonies having the designed mutations in the gene appeared successfully by introducing the plasmids and assisting oligonucleotides to the strains. Most of the plasmids and resultant *leu2*^*−*^ mutants produced in this study will be deposited in several repository organizations.

**Key points:**

*• All-in-one type CRISPR-Cas9 plasmids targeting LEU2 gene were designed for broad application to Saccharomyces sensu stricto group species strains*

*• Application of the plasmids generated leu2 mutants from strains including natural isolates, industrial, and allopolyploid strains*

*• The easy conversion to leu2 mutants permits free access to recombinant plasmids having a LEU2 gene*

**Graphical Abstract:**

**Figure:**
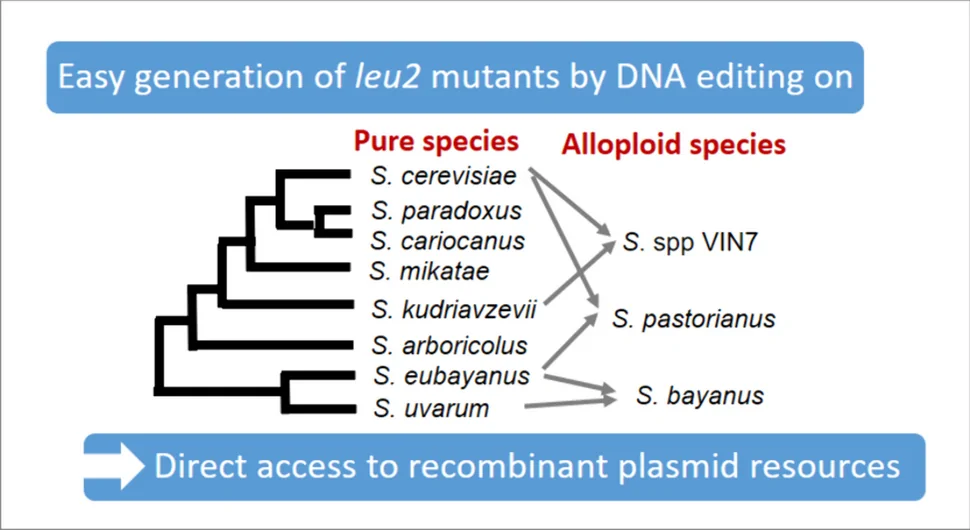

## Introduction

**Fig. 1:**
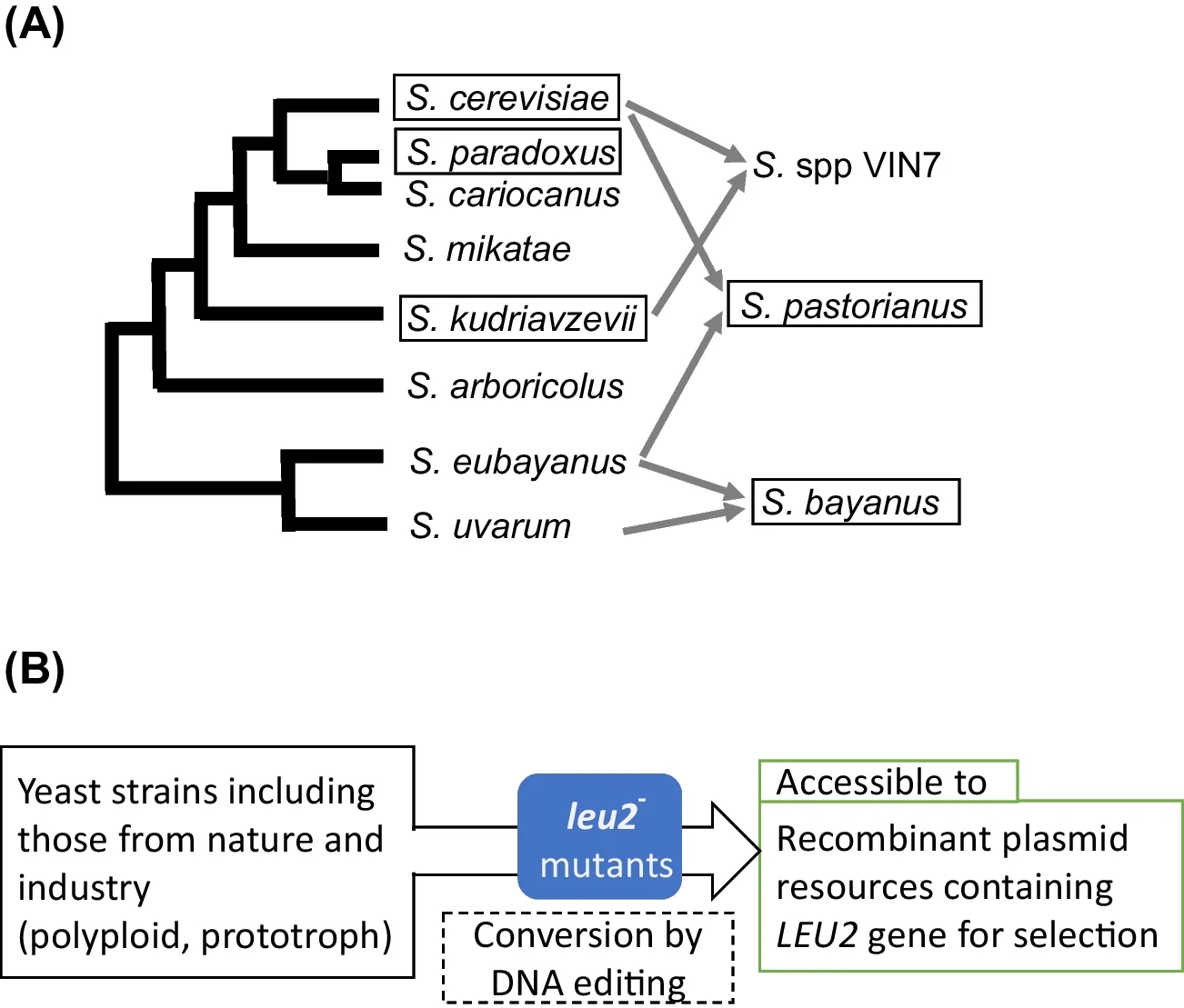
Simple way to convert *Saccharomyces* sensu stricto strains accessible to conventional recombinant plasmid resources. **A** Phylogenetic relationships between *Saccharomyces* sensu stricto group species. A phylogram drawn by Borneman and Pretorius (2015) was adopted with simplification. “Pure” species names are located at the center, whereas alloploid species names are shown on the right side. Allow marks show from which species the ancestor(s) of an alloploid species obtained genomes. Rectangles highlight the species employed for DNA editing experiments in this study. **B** Aims of this study

The yeast *Saccharomyces* sensu stricto complex group comprises eight “pure” species (e.g., *S. cerevisiae*, *S. paradoxus*, *S. kudriavzevii*, *S. eubayanus*) and interspecific hybrid species such as *S. bayanus* and *S. pastorianus* (Borneman and Pretorius 2015), as schematically shown in Fig. 1A. A large number of strains belonging to the yeast group play important roles in the production of foods and beverages in addition to other biotechnological applications (Replansky et al. 2008; Sicard and Legras 2011; Steensels et al. 2014). This number is increasing owing to the employment of new isolates from nature (Hisatomi and Toyomura 2021; Minnaar and den Haan 2023). The species in the group indicate intimate relationships with each other, that is, haploid cells can mate with the opposite mating-type cells belonging to the other species in the group, and the resulting hybrid cells are viable (Toyomura and Hisatomi 2021). However, the yeast group exhibits a large variation in their genomic sequences between species. Synonymous codon similarity % of *S. mikatae*, *S. kudriavzevii*, and *S. bayanus* to *S. cerevisiae* is as low as 55% on average (Cliften et al. 2003), which could ensure the durability of the yeast group in nature and generation of valiant strains for industry.

Since the reliable yeast transformation method was developed (Beggs 1978; Hinnen et al. 1978), every gene of the whole *S. cerevisiae* genome was cloned into various *Escherichia coli*-yeast shuttle vectors and integrative vectors for laboratory strains. For instance, Makanae et al. (2013) developed a series of overexpression plasmids, each of which carries a different protein-coding gene from *S. cerevisiae*. Various recombinant plasmid resources have been deposited in biological resource centers, and the plasmids are distributed on demand. Therefore, it is desirable to apply these plasmid resources to industrial and natural yeast strains for molecular breeding.

Most recombinant plasmids prepared for *S. cerevisiae* contain one of several nutrient synthesis genes (e.g., *LEU2*, *URA3*, and *TRP1*) as the transformant selection marker gene. The corresponding auxotrophic laboratory mutant strains (e.g., *leu2*, *ura3*, and *trp1*) are chosen as the host strain (Akada 2002; Gnugge and Rudolf 2017; Sikorski and Hieter 1989). However, industrial and natural isolate strains generally lack any auxotrophic mutation (prototrophic yeasts) and are mostly diploid and allopolyploid. Therefore, it is difficult to develop auxotrophic mutants suitable for transformation with the conventional recombinant plasmids in such strains. It is possible to add an antibiotic-resistance gene for yeast to the plasmids, but the modification of many substrate plasmids is laborious and time-consuming.

Genome editing of host cell chromosomes is an attractive solution method (Jinek et al. 2012; Raschmanova et al. 2018; Sakuma and Woltjen 2014; Yang and Blenner 2020) to overcome the difficulties in industrial and natural isolate yeast strains. As a convenient and accurate genome editing methodology, the CRISPR-Cas9 system is popular now. A short target sequence (20 nucleotides) in a single-guide RNA (sgRNA) is sufficient for the CRISPR-Cas9 system to generate a double-strand break (DSB) at the target sequence followed by a protospacer adjacent motif (PAM) (Jinek et al. 2012; Ran et al. 2013). In *S. cerevisiae* strains, the application of CRISPR-Cas9 plasmids containing an sgRNA gene results in the formation of mutations at the target locus. Furthermore, a template DNA that covers the target sequence but has some mutations improves the mutant ratio in DNA editing experiments (DiCarlo et al. 2013; Laughery et al. 2015; Raschmanova et al. 2018). Zhang et al. (2014) reported a Cas9 plasmid harboring a neurothricin-resistance gene and a series of sgRNA-expressing plasmids to target *URA3*, *TRP1*, *LEU2*, and *HIS3*, respectively. Successive transformation of an industrial *S. cerevisiae* strain with the Cas9 plasmid and then with one of the sgRNA-expressing plasmids resulted in formation of auxotrophic mutants depending on the guide RNA sequence. Laughery et al. (2015) constructed Cas9 plasmids (pML104 and pML107) containing an sgRNA-expression cassette gene as well as the selection marker gene *LEU2* and *URA3*, respectively. Plasmids simultaneously containing Cas9 and sgRNA genes are categorized as all-in-one type, which is convenient because a single transformation produces mutants.

We aimed to broaden the applicability of the plasmid resources for *Saccharomyces* sensu stricto complex group. In this study, we focused on *LEU2* gene disruption for several reasons. First, *LEU2* has been frequently employed as the selection marker gene in yeast plasmid vectors in conventional plasmid resources. Second, among the auxotrophic mutations apt to the conventional yeast vectors, *leu2* mutants are the most difficult to achieve in polyploid strains by conventional methods because there is no counter-selective characteristic against chemicals, whereas *ura3* mutants are resistant to 5-fluoroorotic acid and *trp1* mutants endure the killing action of 5-fluoroanthranilic acid (Akada 2002; Gnugge and Rudolf 2017; Kitamoto et al. 1990). Here, we show all-in-one type CRISPR-Cas9 plasmids with *LEU2*-gene-targeting guide sequences for application as widely as possible to *Saccharomyces* sensu stricto species. Dissemination of the Cas9 plasmids to yeast strains belonging to five species in the group produced *leu2* mutants at practically desirable frequencies.

## Materials and methods

### Microbial strains, plasmids, and culture conditions

Table 1 lists bacterial and yeast strains and plasmids used in this study.

**Table 1:** Bacterial and yeast strains and plasmids used in this study

(A) Microbial strains			
Strain		Relevant genotype and/or characteristics	Reference or source
*Saccharomyces cerevisiae*			
T55		Laboratory strain, *MATa* prototroph	Tohoyama et al. 1979 (BY29297/NBRP)^1^
T55leu2Δ		*leu2*(547–548, 551) mutant derived from T55	This study (BY29298/NBRP)^1^
T556		Laboratory strain, *MATa/MATa*, prototroph	Cross between T55 and T56, Tohoyama et al. 1979 (BY29299/NBRP)^1^
T556leu2Δ		*leu2*(547–548, 551) mutant derived from T556	This study (BY29300/NBRP)^1^
Kyokai No. 7 (K7)		Rice wine (Sake)-brewing yeast, prototroph	RIB1003/NRIB^2^
K7leu2Δ		*leu2*(547–548, 551) mutant derived from Kyokai No. 7	This study (NRIB)^3^
IS289-1C		*MATa leu2-1 met8-1 aro1B ade8 cdc9*	Lab stock
HD119-3		*MATa trp5 leu1 cyh2*	Lab stock
*S. paradoxus*			
CBS432^T^ (IFO10609^T^)		Type strain, an isolate from *Quercus* spp., prototroph	BY20589/NBRP^1^
CBS432leu2Δ		*leu2*(547–548, 551) mutant derived from CBS432^T^	This study (BY29301/NBRP)^1^
*S. kudriavzevii*			
CBS8840^T^ (IFO1802^T^)		Type strain, an isolate from partially decayed leaf in Japan, prototroph	BY20109/NBRP^1^
CBS8840leu2Δ		*leu2*(245–248) mutant derived from CBS8840^T^	This study (BY29302/NBRP)^1^
*S. bayanus*			
CBS380^T^ (IFO1127^T^)		Type strain, an isolate from turbid beer, prototroph	BY21388/NBRP^1^
CBS380leu2Δ		*leu2*(547–548, 551) mutant derived from CBS380^T^	This study (BY29303/NBRP)^1^
*S. pastorianus*			
W34/70		Lager beer-brewing yeast, prototroph	Hefebank Weihenstephan GmbH, Germany
W34/70leu2Δ		*leu2*(245–248) mutant derived from W34/70	This study (NRIB)^3^
*Escherichia coli*			
JM110		*dam dcm supE44 hsdR17 thi leu rpsL1 lacY galK galT ara tonA thr tsx* *Δ*(*lac-proAB*)/*F'*[*traD36, proAB*^+^*, lacI*^*q*^*, lacZΔM15*]	Yanisch-Perron et al. 1985
HB101		*mcrB mrr hsdS20(rB- mB-) recA13 leuB6 ara-14 proA2 lacY1 galK2 xyl-5 mtl-1 glnV44* λ^−^, Sm^R^	Boyer and Roulland-Dussoix 1969
HB101 RedET		*mcrB mrr hsdS20(rB- mB-) recA13 leuB6 ara-14 proA2 lacY1 galK2 xyl-5 mtl-1 glnV44* λ^−^ (*repA*^*pSC101*^* ori*^pSC101^ *araC P*_*BAD*_*::gbarecA*), Sm^R^ Ap^R^	Lab stock
(B) Plasmids			
Plasmid	Relevant genotype and/or characteristics		Reference or source
Source plasmids to prepare new Cas9 plasmids, a mini plasmid to insert a guide sequence, and a template DNA			
pML104	*ori*^pUC^ *ScURA3 ori_2μ P*_*TDH3*_*::SpCas9 P*_*SNR52*_*::*sgRNA cassette, Ap^R^		Laughery et al. 2015 (#67638/Addgene)^3^
pYAMTr2G	*rep*^pBBR1^ *RB oriT*^pBBR1^ *lacZα KanMX ori_2μ*, Km^R^		Kiyokawa et al. 2023 (BYP10212 /NBRP)^2^
pYAMTrG	*rep*^pBBR1^ *RB oriT*^pBBR1^ *lacZα KanMX ARSH4 CEN6*, Km^R^		Kiyokawa et al. 2023 (BYP9801 /NBRP)^2^
pYAMTrAu	*rep*^pBBR1^ *RB oriT*^pBBR1^ *lacZα AurMX ARSH4 CEN6*, Km^R^		Kiyokawa et al. 2023 (BYP9799 /NBRP)^2^
pBluescript KS ( +)	*ori*^*pUC*^* lacZα*, Ap^R^		Short et al. 1988
Cas9 plasmids and a plasmid to insert guide sequence			
pYAMTr2GCas	*rep*^pBBR1^ *RB oriT*^pBBR1^ *lacZα KanMX ori_2μ P*_*TDH3*_*::SpCas9*, Km^R^		This study (BYP10221/NBRP)^1^
pYAMTrGCas	*rep*^pBBR1^ *RB oriT*^pBBR1^ *lacZα KanMX ARSH4 CEN6 P*_*TDH3*_*::SpCas9*, Km^R^		This study (BYP10222/NBRP)^1^
pBSsgRNA	*ori*^*pUC*^* P*_*SNR52*_*::*sgRNA cassette, Ap^R^		This study (BYP10223/NBRP)^1^
Mini plasmids harboring a guide sequence			
pBSsgScLEU2	*ori*^*pUC*^* P*_*SNR52*_*::*sgRNA cassette with *ScLEU2* guide sequence, Ap^R^		This study
pBSsgSeLEU2	*ori*^*pUC*^* P*_*SNR52*_*::*sgRNA cassette with *SeLEU2* guide sequence, Ap^R^		This study
pBSsgSc-SeLEU2	*ori*^*pUC*^* P*_*SNR52*_*::*sgRNA cassette with *Sc*-*SeLEU2* guide sequence, Ap^R^		This study
Cas9 plasmids harboring a guide sequence			
pYAMTr2GCsgScLEU2	pYAMTr2GC having the guide sequence *ScLEU2*, Km^R^		This study (BYP10224/NBRP)^1^
pYAMTrGCsgScLEU2	pYAMTr2GC having the guide sequence *ScLEU2*, Km^R^		This study (BYP10225/NBRP)^1^
pYAMTr2GCsgSeLEU2	pYAMTr2GC having the guide sequence *SeLEU2*, Km^R^		This study (BYP10226/NBRP)^1^
pYAMTr2GCsgSc-SeLEU2	pYAMTr2GC having the guide sequence *Sc-SeLEU2*, Km^R^		This study (BYP10227/NBRP)^1^
Bacterial-yeast shuttle plasmids having *ScLEU2* to select yeast transformants by Leu^−^ complementation			
YEp351	*ori*^pUC^ *ScLEU2 ori_2μ*, Ap^R^		Hill et al. 1986
YEp351GFP	YEp351 having *P*_*GAL1*_*::GFP*, Ap^R^		This study
pFA6a-kanMX6-PGAL1-GFP	*ori*^pUC^ *KanMx P*_*GAL1*_*::GFP,* Ap^R^		Longtine et al. 1998
Plasmids for trans-kingdom conjugation			
pMz1	*ori*^incQ^ *ScLEU2 ori_2μ mob*^incQ^ *oriT*^incQ^, Ap^R^ Gm^R^		Mizuta et al. 2012
pRH220	*ori*^pUC^ *oriT*^RK2^, *tra*^RK2^ Cm^R^		Mizuta et al. 2012

^1^Serial number in National Bioresource Project (NBRP) budding yeast, Hiroshima, Japan

^2^Serial number at National Research Institute of Brewing (NRIB), Hiroshima, Japan

^3^Deposition communicates with NRIB

Deposition communicates with NRIB and Addgene (Watertown, Massachusetts, USA)

Yeast strains were cultured in YPD medium (1% yeast extract, 2% polypeptone, 2% glucose) at 28 °C. *E. coli* strains were grown in LB medium (1% Bacto tryptone, 0.5% NaCl, 0.5% Bacto yeast extract) at 37 °C.

Yeast strains were cultured also on synthetic media. Synthetic dextrose (SD) medium consists of 2% glucose and 0.67% Bacto yeast nitrogen base without amino acids. Solid media for yeast were prepared by adding 2% agar. Synthetic galactose solid medium is the same with the solid SD medium but contains galactose in place of glucose. To verify auxotrophic mutant phenotypes in recombinant yeasts, colonies were streaked onto solid SD medium and solid SD medium supplemented with 30 μg/ml leucine.

### Plasmid construction

**Fig. 2:**
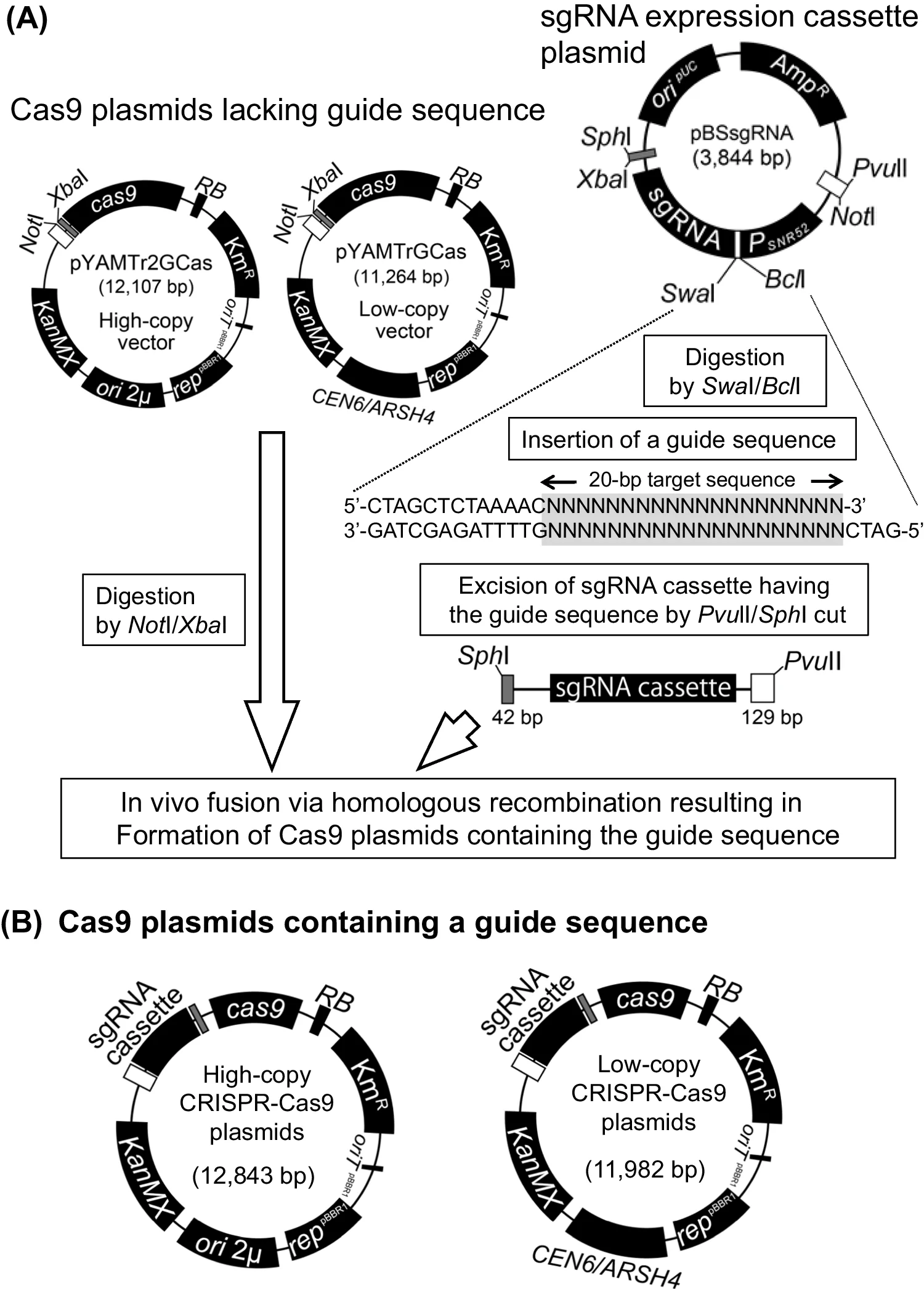
Scheme for preparing CRISPR-Cas9 plasmids harboring a 20-bp guide sequence. **A** Three tool plasmids and processes to construct all-in-one CRISPR-Cas9 plasmids containing a 20-bp guide sequence. The high-copy (*ori 2μ*) yeast vector pYAMTr2GCas and the low-copy (*CEN*/*ARS*) yeast vector pYAMTrGCas lack a guide sequence. The two yeast vectors were linearized by treatment with *Xba*I and *Not*I to accept the guide sgRNA expression cassette. The mini-plasmid pBSsgRNA possesses an sgRNA expression cassette lacking a guide sequence. A ds-oligonucleotide DNA containing a 20-bp guide sequence was ligated with *Bcl*I/*Swa*I-digested pBSsgRNA plasmid to place the target guide sequence on the cassette sgRNA expression cassette. The sgRNA expression cassette with the target guide sequence was excised from the guide-sequence-containing mini-plasmids by digestion with *Pvu*II and *Sph*I. Finally, the *Xba*I/*Not*I-linearized vector plasmids and the *Pvu*II/*Sph*I fragment harboring the sgRNA expression cassette were introduced into either a yeast strain or an *E. coli* strain expressing λ Red recombinase. The vector and guide-containing cassette DNAs were fused together in vivo by homologous recombination at a 42-bp overlapping region (left arm) and a 129-bp overlapping region (right arm). **B** Resultant CRISPR-Cas9 plasmids containing the target guide sequence

An outline of the plasmid structure is shown in Fig. 2.

To build all-in-one type CRISPR-Cas9 plasmids containing the *Cas9* gene and a sgRNA expression cassette with a 20-bp guide sequence, we first constructed two vector plasmids as follows. The *Cas9* gene and a part of the target-lacking sgRNA expression cassette were excised from pML104 (Laughery et al. 2015) as a 5.1-kbp *Eco*RI/*Xba*I DNA fragment. The 5.1-kbp *Eco*RI/*Xba*I DNA fragment was ligated with a high-copy-type yeast Agrobacterium-mediated transformation (AMT) vector pYAMTr2G (Kiyokawa et al. 2023) cleaved by *Eco*RI and *Xba*I (7.0-kbp) and a low-copy-type yeast AMT vector pYAMTrG (Kiyokawa et al. 2023) linearized by *Eco*RI and *Xba*I (6.1-kbp), respectively. The resulting plasmids were named pYAMTr2GCas and pYAMTrGCas, respectively (Fig. 2A).

Second, a mini plasmid harboring the sgRNA expression cassette lacking a target sequence, which was obtained from pML104, was prepared as follows. The target-lacking sgRNA expression cassette was obtained as a 0.9-kbp *Pvu*II/*Xho*I DNA fragment from pML104. The 0.9-kbp *Pvu*II/*Xho*I DNA containing the target-lacking sgRNA expression cassette was ligated with *Eco*RV/*Xho*I-cleaved pBluescript KS ( +) plasmid DNA (Short et al. 1988) to form a 3.9-kbp plasmid named pBSsgRNA (Fig. 2A).

Three 20-bp guide sequences were designed as described in “Guide sequence for CRISPR-Cas9-mediated genome editing.”

Each ds-oligonucleotide guide sequence was inserted into the sgRNA expression cassette of the pBSsgRNA plasmid as follows: The pBSsgRNA plasmid DNA was prepared using a *dam*^−^ *dcm*^−^ *E. coli* strain JM110 (Yanisch-Perron et al. 1985). The pBSsgRNA plasmid was digested with *Swa*I overnight at 25 °C and subsequently treated with *Bcl*I for 4 h at 50 °C. Each of the three sets of complementary oligonucleotide DNA (Table 3 (A1)) containing the 20-bp guide sequence (Table 2 (A)) was ligated with the 3.8-kbp *Swa*I/*Bcl*I-digested pBSsgRNA. The resulting three plasmids were named pBSsgScLEU2, pBSsgSeLEU2, and pBSsgSc-SeLEU2, respectively.

Insertion of the full-length sgRNA expression cassette from each of the three pBSsgRNA derivatives into the two *Cas9* plasmids was performed by in vivo fusion as follows. The *Cas9*-containing plasmids YAMTr2GCas and pYAMTrGCas were linearized by treatment with *Xba*I and *Not*I. The full-length sgRNA expression cassette with a 20-bp guide sequence was taken as a 0.9-kbp *Pvu*II/*Sph*I DNA fragment from pBSsgScLEU2, pBSsgSeLEU2, and pBSsgSc-SeLEU2, respectively. The linearized 12.1-kbp YAMTr2GCas and the 0.9-kbp *Pvu*II/*Sph*I fragment possessing the sgRNA expression cassette were simultaneously introduced into an *E. coli* strain HB101 RedET expressing λ phage recombinase genes *γ*, *β*, and *α*. In the *E. coli* cells, the two DNAs were fused in vivo by homologous recombination at 129-bp and 42-bp overlapping sequences between each end of the two DNAs. The three resulting high-copy-type CRISPR-Cas9 plasmids were named pYAMTr2GCsgScLEU2, pYAMTr2GCsgSeLEU2, and pYAMTr2GCsgSc-SeLEU2, respectively.

Construction of low-copy type all-in-one Cas9 plasmids using pYAMTrGCas was performed as described above, but the in vivo fusion in *E. coli* was replaced with that in yeast strain T55. The resulting low-copy-type CRISPR-Cas9 plasmids were named pYAMTrGCsgScLEU2, pYAMTrGCsgSeLEU2, and pYAMTrGCsgSc-SeLEU2, respectively.

YEp351_PgalGFP was prepared by ligation between *Sal*I- and *Bam*HI-digested YEp351 and 1.5 kbp fragment from *Sal*I- and *Bgl*II-digested pFA6a-kanMX6-PGAL1-GFP.

### Guide sequence for CRISPR-Cas9-mediated genome editing

We selected 20-bp guide sequences in the target *LEU2* gene as follows: Genome sequences of 50 *S. cerevisiae* strains were retrieved from the *Saccharomyces* genome database ((Cherry et al. 2012); https://www.yeastgenome.org). To identify common sequences among 50 *S. cerevisiae* strains in each target gene, multiple sequence alignment was performed using the MAFFT program version 7.463 (Katoh and Standley 2013). Target sequence candidates with low off-target effects were searched using CRISPRdirect (Naito et al. 2015) and CRISPOR (Concordet and Haeussler 2018) programs. Highly conserved regions in the *LEU2* gene were used as the query sequence. We examined the applicability of the target sequence candidates to *Saccharomyces* sensu stricto species using the nucleotide blast program and chose guide sequences (Table 2) to make the applicability as wide as possible.

**Table 2:** Target sequence in *LEU2* gene for CRISPR-Cas9

(A) Guide sequences designed				
Name	20-bp Guide sequence + PAM	Position in ORF	Cas9 sgRNA expression plasmid harboring each guide sequence	
ScLEU2	AAGGACCAAATAGGCAATGGTGG	569–547	pYAMTrGCScLEU2 pYAMTr2GCScLEU2	
SeLEU2	AAGGACCA**G**ATAGG**T**AATGGTGG	569–547	pYAMTr2GCSeLEU2	
Sc-SeLEU2	TGCTGTGGGTGGTCCTAAATGGG	225–247	pYAMTr2GCSc-SeLEU2	
(B) Sequence difference from the guide sequences				
Species, strain	ScLEU2 (SeLEU2) + PAM	Type	Sc-SeLEU2 + PAM	GenBank assembly accession
* S. cerevisiae* S288c	AAGGACCAAATAGGCAATGGTGG	Sc	TGCTGTGGGTGGTCCTAAATGGG	GCA_000146045.2
* S. paradoxus* CBS432^T^	AAGGACCAAATAGGCAATGGTGG	Sc	TGCTGTGGGTGGTCCTAAATGGG	GCA_002079055.1
* S. cariocanus* NCYC 2890^ T^	(Data absent)		(Data absent)	(Data absent)
* S. mikatae* IFO1815^T^	AAGGACCAAATAGGCAATGGTGG	Sc	TGC**A**GT**A**GGTGGTCCTAAATGGG	GCA_000167055.1
* S. kudriavzevii* CBS8840^T^	(Data absent)		(Data absent)	GCA_00167075.2
* S. arboricolus* H-6^ T^	AAGGACCAAATAGGCAATGGTGG	Sc	TGCTGT**A**GGTGGTCCTAAATGGG	GCA_000292725.1
* S. eubayanus* CBS12357^T^	AAGGACCA**G**ATAGG**T**AATGGTGG	Se	TGCTGTGGGTGGTCCTAAATGGG	GCA_001515405.2
* S. uvarum* CBS7001	AAGGACCA**G**ATAGG**T**AATGGTGG	Se	TGCTGTGGGTGGTCCTAAATGGG	GCA_027557585.1
* S. pastorianus* CBS1513^T^	AAGGACCAAATAGGCAATGGTGG	Sc	TGCTGTGGGTGGTCCTAAATGGG	GCA_013180785.1 (scaffold1263_cov98)
	AAGGACCA**G**ATAGG**T**AATGGTGG	Se	TGCTGTGGGTGGTCCTAAATGGG	GCA_013180785.1 (scaffold184_cov57)
* S. bayanus* CBS380^T^	AAGGACCA**G**ATAGG**T**AATGGTGG	Se	TGCTGTGGGTGGTCCTAAATGGG	GCA_013180675.1

A bold underlined face letter indicates a nucleotide sequence different from that of *S. cerevisiae*

### DNA transformation in yeast

The lithium acetate (LiAc) yeast transformation method was performed according to a protocol described by Gietz and Schiestl (2007) with minor modifications. Yeast cells (1.9 × 10^7^ colony forming unit (CFU)) were suspended in a solution containing 100 ng of circular plasmid DNA, LiAc, polyethylene glycol (PEG) 4000, and carrier DNA and incubated for 40 min at 42 °C.

Before selection using G418, the yeast cells were then cultured in liquid YPD medium at 28 °C for 2 h (for experiments using laboratory strains only) or 3.5 h. After the cultivation, yeast cells were collected and spread on solid YPD medium containing 400 μg/ml G418. To enhance the mutant frequency, 50 pmol of a hybridized 90-base oligonucleotide DNA pair (which were named as dsOligo DNAs as listed in Table 3 (B)) was added as the template DNA together with the CRISPR-Cas9 plasmid DNA to the LiAc/PEG 4000/carrier DNA solution.

**Table 3:** List of oligonucleotides used in this study

(A) Oligonucleotides for preparation of guide sequences and of template DNAs			
Oligonucleotide name	Sequence (5’-3’)		Application
(A1) Complementary pairs for preparation of a guide sequence			Resultant plasmid
ScLEU2sgRNA_Fw	GATCAAGGACCAAATAGGCAATGGGTTTTAGAGCTAG		pBSsgScLEU2
ScLEU2sgRNA_Rv	CTAGCTCTAAAACCCATTGCCTATTTGGTCCTT		
SeLEU2sgRNA_Fw	GATCAAGGACCAGATAGGTAATGGGTTTTAGAGCTAG		pBSsgSeLEU2
SeLEU2sgRNA_Rv	CTAGCTCTAAAACCCATTACCTATCTGGTCCTT		
Sc-SeLEU2sgRNA_Fw	GATCTGCTGTGGGTGGTCCTAAATGTTTTAGAGCTAG		pBSsgSc-SeLEU2
Sc-SeLEU2sgRNA_Rv	CTAGCTCTAAAACATTTAGGACCACCCACAGCA		
(A2) Complementary pairs for preparation of template DNAs			Resultant template DNA
D-ScLEU2_Fw	AGAATCACAAGAATGGCCGCTTTCATGGCCCTACAACATGAGACTATTGCC TATTTGGTCCTTGGATAAAGCTAATGTTTTGGCCTCTTC		dsOligo DNA (ScLEU2)
D-ScLEU2_Rv	GAAGAGGCCAAAACATTAGCTTTATCCAAGGACCAAATAGGCAATAGTCT CATGTTGTAGGGCCATGAAAGCGGCCATTCTTGTGATTCT		
D-SeLEU2_Fw	AGAATTACAAGAATGGCTGCGTTCATGGCACTACAACACCAAACTATTAC CTATCTGGTCCTTGGATAAAGCCAATGTTTTGGCCTCATC		dsOligo DNA (SeLEU2)
D-SeLEU2_Rv	GATGAGGCCAAAACATTGGCTTTATCCAAGGACCAGATAGGTAATAGTTTG GTGTTGTAGTGCCATGAACGCAGCCATTCTTGTAATTCT		
D-Sc-SeLEU2_Fw	AGAAGGCTGATGCCGTTTTGTTAGGTGCTGTGGGTGGTCCTAAATTACCGGTA GTGTTAGACCTGAACAAGGTTTACTAAAAATCCGTAA		dsOligo DNA (Sc-SeLEU2)
D-Sc-SeLEU2_Rv	TTACGGATTTTTAGTAAACCTTGTTCAGGTCTAACACTACCGGTAATTTAGG ACCACCCACAGCACCTAACAAAACGGCATCAGCCTTCT		
(B) Primers for PCR to prepare sequence template and for sequencing			
Oligonucleotide name	Sequence (5′-3′)	PCR and sequence target	Yeast species
F-PCR_LEU2-UP_Fw	GTACCGGTAGTGTTAGAC	*ScLEU2*	*S. cerevisiae*, *S. paradoxus*
F-PCR_LEU2-DO_Rv	GTTCGTACAAACCAAATGC		
Seu_LEU2_Fw2	GGTCAAGAAATCACTGAGG	*SeLEU2*	*S. bayanus*
Seu_LEU2_Rv2	CCAATGAACCAGGAATACAA		
SkuLEU2_Fw	GAAGCAATTAAGGTTCT	*Sc-SeLEU2*	*S. kudriavzevii*
SkuLEU2_Rv	CTCCCACTAACTCTCTAAC		
F-PCR_LEU2-UP_Fw2^1^	GAAGCCATTAAGGTTCT	*Sc-SeLEU2*	*S. pastorianus*
Sc_LEU2_Rv2^1^	AAGGAACCTGGGATAACG		
Seu_LEU2_Rv2^1^	CCAATGAACCAGGAATACAA		

^1^F-PCR_LEU2-UP_Fw2 anneals with *Sc*-*LEU2* target sequence and with *Se*-*LEU2* target sequence, while Sc_LEU2_Rv2 and Se_LEU2_Rv2 specifically hybridizes with the targets *ScLEU2* and *SeLEU2*, respectively

Transformation frequency was represented as the number of transformants per μg of plasmid DNA per output viable cell number after LiAc/PEG 4000 treatment.

### Trans-kingdom conjugation to yeast

A mobilizable *LEU2* plasmid pMz1was transferred from a donor *E. coli* strain HB101 that harbors pMz1 and a helper plasmid pRH220 as shown by Mizuta et al. (2012). After co-incubation between a *leu2* mutant yeast and the donor *E. coli* cells, the cell mixture was spread onto SD solid medium, which allows selective growth of yeast cells that obtained pMz1.

### Nucleotide sequence determination

The DNA sequencing was determined using the Sanger method by Eurofin Co. (Tokyo, Japan). Template DNA sequencing reaction was prepared by PCR amplification using primer sets listed in Table 3 (B). In *leu2* mutants in aneuploid strains, each of two *leu2* genes was amplified using allele-specific primer sets shown in Table 3 (B) and sequenced respectively.

### Statistical analysis

Statistical analysis was performed using the R program version 4.0.2 and its expansion package (https://www.R-project.org/). Each datum in a table represents a set of a mean value ± its standard deviation (SD). Individual methods and experimental replicates for statistical comparisons are presented in each table.

## Results

### Construction of all-in-one CRISPR-Cas9 plasmids for conversion of prototrophic yeast strains to leu2 mutant strains

Figure 2 depicts a scheme to construct all-in-one CRISPR-Cas9 plasmids. Using a Cas9 expression gene and sgRNA expression gene cassette from pML104 (Laughery et al. 2015), we prepared three tool plasmids, namely, a small plasmid pBSsgRNA and two CRISPR-Cas9 vector plasmids (pYAMTr2GCas and pYAMTrGCas), which lacked the target sequence. A 20-bp guide sequence for Cas9 scission was inserted into the sgRNA expression gene cassette in pBSsgRNA, and the cassette with the guide sequence was then moved to one of the two CRISPR-Cas9 vector plasmids. The vector plasmid pYAMTr2GCas is a high-copy and pYAMTrGCas is a low-copy replication type, because the former contains *ori_2μ* and the latter harbors *CEN*/*ARS* (Sikorski and Hieter 1989). Because half of the two vectors were derived from pYAMTrG and pYAMTr2G (Kiyokawa et al. 2023), respectively, they harbored the G418 resistance gene cassette *KanMX* to enable transformant selection for prototrophic yeast strains and contained a right border sequence (*RB*), an overdrive sequence, and the pBBR1 replication gene, which as a whole permit mobilization by the AMT method.

Figure 1A illustrates the phylogenetic relationship of yeast species among *Saccharomyces* sensu stricto group. As listed in Table 2 (A), we designed three 20-bp guide sequences for DNA editing at *LEU2* gene, aiming as broadly as possible among *Saccharomyces* sensu stricto species strains. In DNA databases, *LEU2* gene sequence was available for eight species in the group (Table 2 (B)). The three guide sequences matched well with the sequences in the database and were equipped to the two Cas9 vector plasmids, as listed in Table 2 (A).

In this study, we used seven strains belonging to five yeast species. Most of these strains are natural and industrial strains. As a pilot application with a pedestal purpose, we examined the basic behavior of yeast toward the CRISPR-Cas9 plasmids and template DNAs using two *S. cerevisiae* laboratory strains.

### High yield of leu2 mutant strains from *S. cerevisiae* laboratory strains

The two all-in-one type plasmids pYAMTrGCsgScLEU2 and pYAMTr2GCsgScLEU2 contain a 20-bp guide sequence for *S. cerevisiae LEU2* gene (Table 2 (A)). The former plasmid is of the multi-copy type and the latter is of the low-copy type. Introduction of the two plasmids into the haploid laboratory strain T55 generated prototrophic (Leu^+^) colonies and leucine-auxotrophic (Leu^−^) colonies. As shown in Table 4, the ratio of Leu^−^ colonies was approximately 90%, irrespective of the plasmid copy difference. The diploid laboratory strain T556 produced fewer recombinant colonies than the haploid strain. The ratio of Leu^−^ colonies by the diploid strain was around 50%, which is a half the ratio of the haploid strain.

**Table 4:** *LEU2* gene disruption in haploid and diploid *S. cerevisiae* laboratory strains

Yeast strain		Template DNA^1^	G418^R^ recombinant colony number		Transformation efficiency (× 10^−4^)		Leu^*−*^ mutant ratio^3^ (%)	
(A) pYAMTr2GCsgScLEU2 (high copy)								
Haploid	T55	dsOligo DNA (ScLEU2)	3930 ± 177	b^2^	1.9 ± 0.6	b^2^	118/120	98
		ー	78 ± 11	a	0.04 ± 0.01	a	91/101	90
Diploid	T556	dsOligo DNA (ScLEU2)	1840 ± 856	b	1.2 ± 0.7	b	116/120	97
		ー	10 ± 4	a	0.005 ± 0.0006	a	9/23	39
(B) pYAMTrGCsgScLEU2 (low copy)								
Haploid	T55	dsOligo DNA (ScLEU2)	301 ± 145	b	0.2 ± 0.1	b	155/158	98
		ー	16 ± 3	a	0.01 ± 0.002	a	50/53	94
Diploid	T556	dsOligo DNA (ScLEU2)	178 ± 78	b	0.1 ± 0.06	b	119/126	94
		ー	7 ± 5	a	0.004 ± 0.002	a	12/20	60

Haploid laboratory strain T55 and diploid laboratory strain T556 were transformed with pYAMTr2GCsgScLEU2 (A) and pYAMTrGCsgScLEU2 (B) using LiAc method. After LiAc/PEG treatment, the yeast cells were cultured in liquid YPD medium at 28 °C for 2 h

^1^An annealed pair of oligonucleotide DNAs (dsOligo DNA), which fit the assumed double-strand-break end regions at the target in *ScLEU2*, was applied as the template DNA together with the all-in-one CRISPR-Cas9 plasmids to the yeast strains. See Table 3 (B)

^2^Different letters indicate significant differences (*P* < 0.05) according to the Tukey–Kramer test (*n* = 3–4)

^3^Ratio of leucine-auxotrophic colonies per G418^R^ resistant colonies

We prepared ds-oligonucleotide DNA (Sc), which is a hybridization of two complementary 90-base sequences that match the target region of *ScLEU2* but lacks two nucleotides in the PAM sequence (Table 3 (A2)). Addition of the ds-oligo DNA markedly increased the number of recombinant colonies by more than tenfold, and the Leu^−^ ratio was up to 98% in the haploid strain and approximately 95% in the diploid strain (Table 4).

**Fig. 3:**
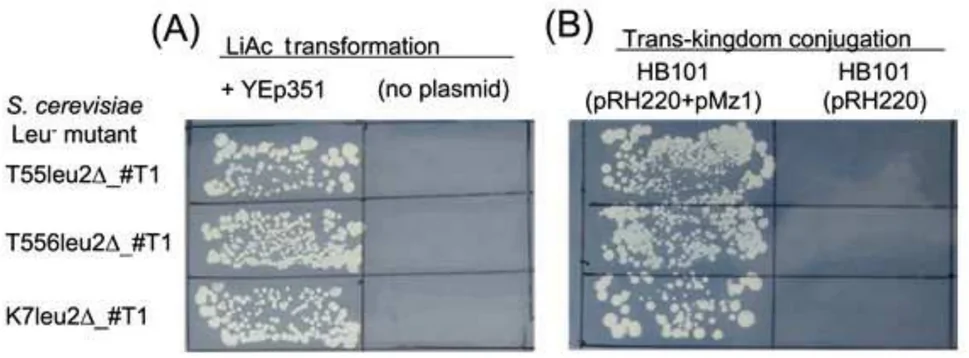
Rescue of Leu^−^ phenotype by introduction of *ScLEU2* gene. **A** LiAc transformation with YEp351, which harbors *ScLEU2*. **B** Trans-kingdom conjugation using a donor *E. coli* strain HB101 containing a helper plasmid pRH220 and a *ScLEU2*-containing mobilizable plasmid pMz1

**Fig. 4:**
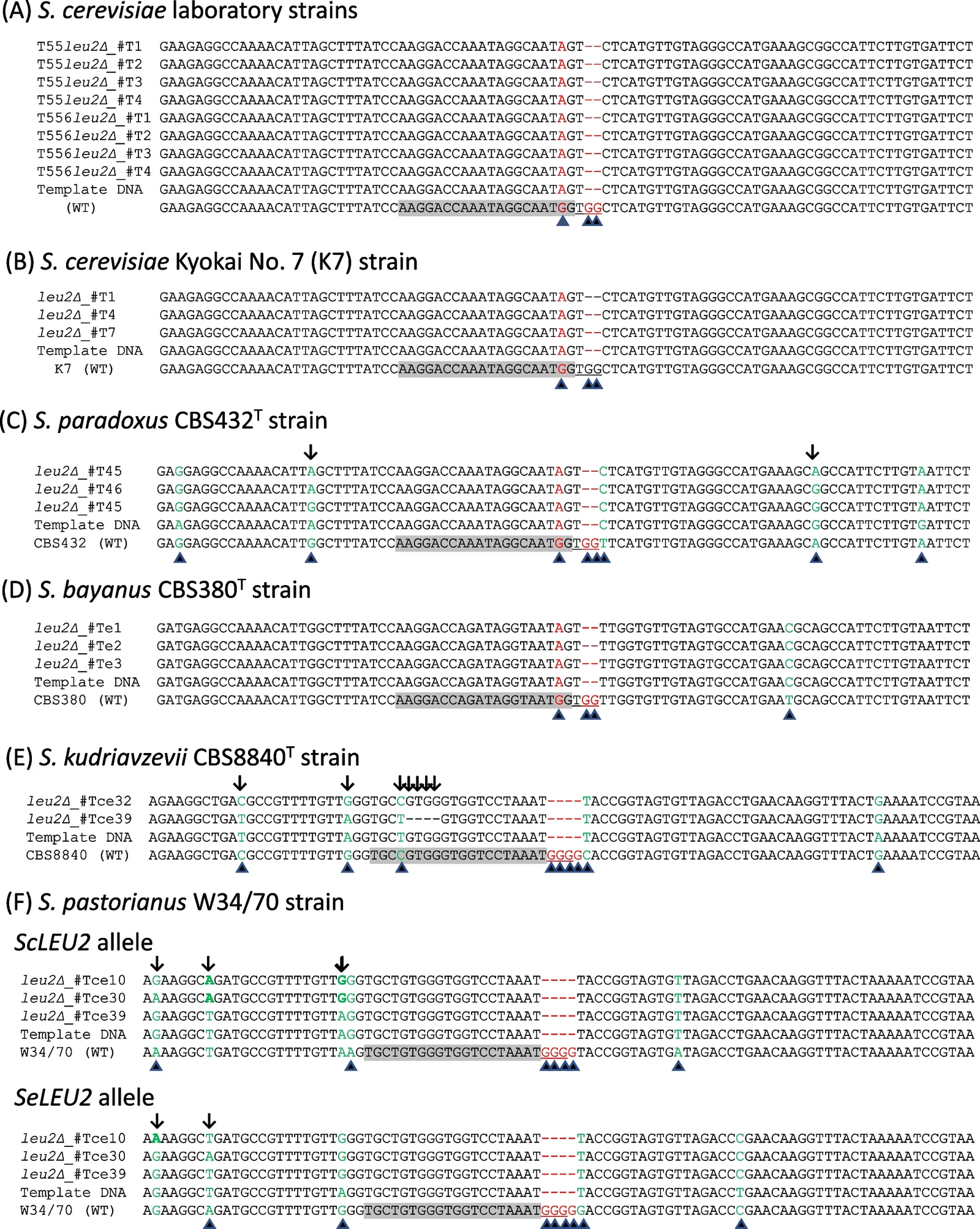
Verification of *leu2* mutations by nucleotide sequence analysis. A template 90-bp oligonucleotide DNA sequence was aligned with the corresponding wild-type (WT) and mutant gene sequences. Shade boxes and underlines indicate the 20-bp guide sequence and PAM, respectively. Arrows and filled triangles indicate nucleotide polymorphisms among *leu2* mutant strains and between wild-type and template DNA sequences, respectively. **A** Four mutants derived from *S. cerevisiae* strain T55 and four mutants from the diploid strain T556. **B** Three mutants of the sake-producing strain *S. cerevisiae* Kyokai No. 7 (K7). **C** Three mutants of *S. paradoxus* strain CBS432^T^. **D** Three mutants of *S. bayanus* strain CBS380^T^. **E** Two mutants from *S. kudriavzevii* strain CBS8840^T^. **F** Three mutants of the beer-producing *S. pastorianus* strain W34/70

The mutation(s) that caused the Leu^−^ phenotype were located at the *LEU2* locus. First, leucine auxotrophy of the Leu^−^ mutant recombinant colonies was rescued by LiAc transformation with YEp351 (Fig. 3A) and by trans-kingdom conjugation with a donor *E. coli* HB101 harboring a mobilizable *ScLEU2* plasmid pMz1 and a helper plasmid pRH220 (Fig. 3B). Second, crossing haploid Leu^−^ colonies with an opposite mating-type *leu2* mutant tester strain IS289-1C failed to complement the Leu^−^ phenotype, while crossing with an opposite mating-type *leu1* mutant tester strain HD119-3 complemented the Leu^−^ phenotype (data not shown). Finally, DNA sequencing analysis verified that the mutation is present at the target sequence in *LEU2* gene as shown in Fig. 4A. Three haploid and three diploid mutants had a sequence identical to the template ds-oligonucleotide DNA sequence.

### Generation of leu2 mutants from a sake (rice-wine) brewing strain and a *S. paradoxus* strain

We applied the high-copy plasmid pYAMTr2sgScLEU2 and the template ds-oligonucleotide DNA (Sc) to five yeast strains, which consisted of industrial and natural isolate strains.

Sake-producing *S. cerevisiae* strain Kyokai No. 7 (K7) is a diploid (Akao et al. 2011). The strain showed a much lower Leu^−^ mutant ratio (19%) than the diploid laboratory strain T556 (100%). Conversely, K7 strain yielded a sevenfold larger number of G418^R^ recombinant colonies than T556 (Table 5). We examined three Leu^−^ mutants of the K7 strain using DNA sequencing analysis at the *LEU2* locus. All three K7 mutants exhibited a sequence identical to the template ds-oligonucleotide DNA sequence (*Sc*) (Fig. 4B).

**Table 5:** Leu^−^ mutant formation induced by CRISPR-Cas9 plasmids in yeasts belonging to five *Saccharomyces* sensu stricto species

Yeast strain	CRISPR Cas9 plasmid having target sequence type^1^ in *LEU2* gene	Template DNA^2^	G418^R^ recombinant colony number	Transformation efficiency (× 10^−5^)	Leu^−^ mutant ratio (%)	
*S. cerevisiae*						
T556	*ScLEU2*	dsOligo DNA (ScLEU2)	547 ± 119	1.5 ± 0.3	40/40	100
	*SeLEU2*	dsOligo DNA (SeLEU2)	(1.1 ± 0.3) × 10^4^	38 ± 19	0/120	0
	*Sc-SeLEU2*	dsOligo DNA (Sc-SeLEU2)	560 ^3^	1.9 ^3^	6/6 ^3^	100 ^3^
	(No plasmid)	(No DNA)	Not detected	–	–	–
Kyokai No. 7	*ScLEU2*	dsOligo DNA (ScLEU2)	4063 ± 1137	22 ± 8.8	23/120	19
	(No plasmid)	(No DNA)	Not detected	−	−	−
*S. paradoxus*						
CBS432^T^	*ScLEU2*	dsOligo DNA (ScLEU2)	132 ± 46	0.8 ± 0.3	112/120	93
	(No plasmid)	(No DNA)	Not detected	–	–	–
*S. bayanus*						
CBS380^T^	*ScLEU2*	dsOligo DNA (ScLEU2)	751 ± 57	6.9 ± 1.1	0/168	0
	*SeLEU2*	dsOligo DNA (SeLEU2)	111 ± 53	0.9 ± 0.4	120/120	100
	*Sc-SeLEU2*	dsOligo DNA (Sc-SeLEU2)	537 ^3^	5.5 ^3^	40/40 ^3^	100 ^3^
	(No plasmid)	(No DNA)	Not detected	–	–	–
*S. pastorianus*						
W34/70	*ScLEU2*	dsOligo DNA (ScLEU2)	272 ± 126	1.9 ± 0.8	0/168	0
	*SeLEU2*	dsOligo DNA (SeLEU2)	303 ± 228	2.9 ± 2.6	0/120	0
	*Sc-SeLEU2*	dsOligo DNA (Sc-SeLEU2)	1106 ± 262	11 ± 3.4	36/224	16
	(No plasmid)	(No DNA)	Not detected	–	–	–
*S. kudriavzevii*						
CBS8840^T^	*ScLEU2*	dsOligo DNA (ScLEU2)	69 ± 50	1.9 ± 1.6	0/117	0
	*SeLEU2*	dsOligo DNA (SeLEU2)	435 ± 339	12 ± 11	0/120	0
	*Sc-SeLEU2*	dsOligo DNA (Sc-SeLEU2)	435 ± 174	10 ± 7.9	20/120	9
	(No plasmid)	(No DNA)	Not detected	–	–	–

Yeast strains were transformed with a combination of a CRISPR Cas9 plasmid and its corresponding template DNA: pYAMTr2GCsgScLEU2 plasmid and a template ds-oligonucleotide DNA (*Sc*); pYAMTr2GCsgSeLEU2 and ds-oligonucleotide DNA (*Se*); pYAMTr2GCsgSc-SeLEU2 and ds-oligonucleotide DNA (*Sc*-*Se*)

To yield larger number of recombinant colonies from *Saccharomyces* sensu stricto yeasts, cultivation after LiAc/PEG treatment was extended to 3.5 h (see “Materials and methods”)

^1^See Table 2 (A)

^2^See Table 3 (B) and footnote #3 in Table 4

^3^Data from one time experiment

*S. paradoxus* CBS432^T^ is a diploid strain. As shown in Fig. 2B, CBS432^T^ strain’s *LEU2* gene has 20-bp guide and PAM sequences identical to those of the *ScLEU2* gene, whereas there were single nucleotide polymorphisms (SNPs) near the target sequence in *S. paradoxus LEU2* compared with *ScLEU2* (data not shown). As indicated in Table 5, the CBS432^T^ strain showed a high Leu^−^ mutant ratio (93%). We analyzed the *LEU2* sequence in three CBS432^T^ Leu^−^ mutant strains. All three mutants contained one nucleotide substitution in the target sequence and lacked PAM, which appears to reflect an event of replacement by double crossovers with the template ds-oligonucleotide DNA (*Sc*) (Fig. 4C). In contrast, the three CBS432^T^ *leu2* mutant strains showed small differences from the template DNA in sequences surrounding the target sequence, which is likely to show crossover positions near the scission site between the template ds-oligonucleotide DNA and the chromosomal *LEU2* gene in the *S. paradoxus* strain (Fig. 4C).

In contrast to the *S cerevisiae* and *S. paradoxus* strains, application of the same plasmid and the template ds-oligonucleotide DNA produced no auxotrophic mutant in *S. kudriavzevii* CBS8840^T^, *S. bayanus* CBS380^T^, and *S. pastorianus* W34/70 (Table 5). This result for *S. bayanus* and *S. pastorianus* coincides with the estimation based on their *LEU2* gene sequences in the database (Table 2 (B)), whereas no data were available for *S. kudriavzevii*.

### Formation of leu2 mutants from S. bayanus using a guide sequence suitable for S. eubayanus-type LEU2

As shown in Fig. 1A, S*. bayanus* CBS380^T^ is an allodiploid strain whose hybrid genome originated from *S. eubayanus* and *S. uvarum*, whereas *S. pastorianus* W34/70 is an allodiploid strain with an amalgam genome derived from *S. eubayanus* and *S. cerevisiae* (Borneman and Pretorius 2015; de la Cerda Garcia-Caro et al. 2022). The all-in-one CRISPR-Cas9 plasmid pYAMTr2GCsgSeLEU2 contains a guide sequence (SeLEU2) suitable for *S. eubayanus LEU2* gene as the target.

The *S. bayanus* strain CBS380^T^ was transformed simultaneously with pYAMTr2GCsgSeLEU2 and a template ds-oligonucleotide DNA (Se), which is similar to the template ds-oligonucleotide DNA (Sc) but suits *S. eubayanus LEU2* (Fig. 4D). As indicated in Table 5**,** the CBS380^T^ strain produced Leu^−^ colonies upon the transformation. Its Leu^−^ ratio was 100%. DNA sequencing analysis verified that three *S. bayanus* Leu^−^ mutants had a sequence identical to the ds-oligonucleotide DNA (Se) sequence in the target *LEU2* locus (Fig. 4D).

Conversely to *S. bayanus*, and accordingly with the estimation shown in Table 2 (B), *S. pastorianus*, *S. kudriavzevii*, and *S. cerevisiae* strains failed to generate any Leu^−^ mutants by transformation with pYAMTr2GCsgSeLEU2 and its template (Table 5).

### Conversion of S. pastorianus and S. kudriavzevii strains to leu2 mutants using a guide sequence common to *S. cerevisiae*-type and S. eubayanus-type LEU2

The all-in-one CRISPR-Cas9 plasmid pYAMTr2GCsgSc-SeLEU2 contains a 20-bp guide sequence (*Sc*-*Se LEU2* in Table 2), which is highly conserved between *Sc*-type and *Se*-type *LEU2*.

The *S. pastorianus* strain W34/70 was transformed with a mixture of pYAMTr2GCsgSc-SeLEU2 and its corresponding template ds-oligonucleotide DNA (*Sc*-*Se*). The resulting recombinant W34/70 colonies exhibited a Leu^−^ mutant ratio 17% (Table 5). The same mixture was also applied to *S. kudriavzevii* strain CBS8840^T^as well as to *S. cerevisiae* T556 and *S. bayanus* CBS380^T^ strains. This treatment generated Leu^−^ colonies. The Leu^−^ ratio among recombinant colonies of CBS8840^T^ was 7%, whereas that of T556 and CBS380^T^ was 100% (Table 5).

DNA sequencing analyses revealed that all Leu^−^ mutants from W34/70 and CBS8840^T^ strains had a four-nucleotide deletion surrounding PAM, which is likely due to a double-crossover with the template DNA at the target *LEU2* locus (Fig. 4E, F). Leu^−^ mutants from *S. pastorianus* strain W34/70 had some nucleotide polymorphisms, which were probably a consequence of homologous recombination between chromosomal *Sc*-type and *Se*-type *LEU2* alleles at the target locus (Fig. 4F). One CBS8840^T^ Leu^−^ mutant strain (#Tce39) contained an additional four-nucleotide deletion in the target sequence (Fig. 4E). These results support our assumption that the pYAMTr2GCsgSc-SeLEU2 plasmid, which has the guide sequence Sc-SeLEU2 most common to both *Sc*-type and *Se*-type *LEU2* genes, is applicable to a broad range of *Saccharomyces* sensu stricto yeasts to generate *leu2* mutant strains.

### Easy removal of all-in-one CRISPR-Cas9 plasmid from recombinant leu2 mutants

After DNA editing by plasmid transformation, the plasmid DNA needs to be removed from the resultant mutant transformants for subsequent studies. We analyzed the loss frequency of a resident CRISPR-Cas9 plasmid from recombinant *leu2* mutants. First, we cultured *leu2* mutant strains induced by pYAMTr2GCsgScLEU2 derived from *S. cerevisiae* T556 and *S. paradoxus* CBS432^T^ in liquid YPD medium (without G418) for 24 h and then established colonies on solid YPD medium. Sensitivity to G418 was examined for each colony. All 40 T556- and CBS432^T^-derived colonies were sensitive to G418, indicating that the resident CRISPR-Cas9 plasmid is easily removable.

One *leu2* mutant from each of the seven strains was treated as described above. Consequently, G418-sennsitive colonies were obtained easily. PCR analysis further confirmed the absence of the Cas9 plasmid in a G418^S^ *leu2* mutant colony of the respective strains. The *leu2* mutant strains free of the Cas9 plasmid are available in two public depositories (Table 1 B).

### Expression of a foreign gene by transformation of resulting leu2 mutants

**Fig. 5:**
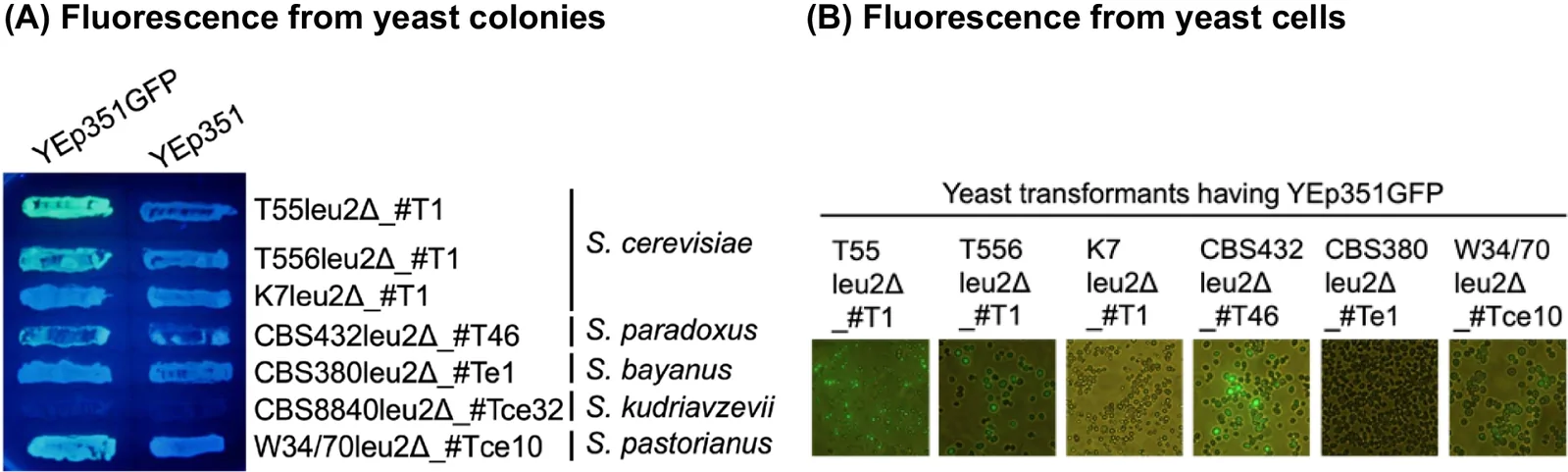
Introduction of a green fluorescent protein gene in yeast *leu2* mutants. **A** Yeast *leu2* mutants transformed with YEp351 and its derivative plasmid (YEp351GFP) containing a P_GAL1_::*gfp* gene were grown on synthetic galactose solid medium. Yeast colonies were irradiated with 365 nm LED light. **B** The transformed yeast cells harboring YEp351_P_GAL1_GFP were observed under blue light excitation

The *leu2* mutants were transformed with a *LEU2* plasmid YEp351GFP, which contains a green fluorescent protein gene that is located just downstream of the promoter of *ScGAL1*. As shown in Fig. 5, *leu2* mutants derived from *S. cerevisiae* T55 and T556, *S.* *paradoxus* CBS432^T^, *S.* *pastorianus* W34/70 emitted green fluorescence when transformed with YEp351GFP and grew on the galactose medium, while those with the vacant vector plasmid YEp351 did not indicate the fluorescence. Mutants from *S. cerevisiae* K7 and *S. bayanus* CBS380^T^ did not emit the fluorescence. The *S.* *kudriavzevii* mutants as well as *S.* *kudriavzevii* CBS8840^T^ did not grow on the galactose medium. These results suggest that the *S. paradoxus* and *S.* *pastorianus* strains possess a regulatory mechanism similar with that of the *S. cerevisiae* galactose regulon, while the *S. cerevisiae* K7 and *S. bayanus* CBS380^T^ strains have a different mechanism.

### Applicability prediction of the all-in-one plasmids to other yeast species

In this study, we did not handle strains belonging to five “pure species,” namely, *S. cariocanus*, *S. mikatae*, *S. arboricolus*, *S. eubayanus*, and *S. uvarum*. However, it is easy to predict that *S. eubayanus* and *S. uvarum* are good substrates for *LEU2* gene disruption by the plasmids containing the *S. eubayanus* type guide (Se), because 100% Leu^−^ ratio was exhibited (Table 5) by the allodiploid *S. bayanus* strain CBS380^T^, whose genome was derived from *S. eubayanus* and *S. uvarum* (Borneman and Pretorius 2015). Furthermore, the DNA database supplied *LEU2* gene sequences for *S. eubayanus* and *S. uvarum* (Table 2 (B)). The sequence data indicated a perfect match to the guide sequence Se-SeLEU2 (Table 2 (B)). Similarly, the *LEU2* sequences of *S. mikatae* and *S. arboricolus* suited exactly to the guide sequence SeLEU2. These data forecast the suitability of the Cas9 plasmids for the five remaining species.

## Discussion

In this study, we prepared *leu2* mutants from seven prototrophic yeast strains belonging to five species of the *Saccharomyces* sensu stricto group by DNA editing. Leu^−^ mutant ratios varied among the yeast strains. Three diploid strains, namely, one *S. cerevisiae* laboratory strain T556 and the type strains of *S. paradoxus* and *S. bayanus*, achieved an almost 100% Leu^−^ mutant ratio (Table 5). Contrary to the high efficacy of the three diploid strains, *S. cerevisiae* Kyokai No. 7 (K7), *S. kudriavzevii* CBS8840^T^, and *S. pastorianus* W34/70 exhibited lower Leu^−^ ratios, 9–19% (Table 5). Despite the unexpectedly low ratio level in the three strains, it was easy to find the target *leu2* mutants because the discrimination between Leu^+^ and Leu^−^ was a facile work for the number of colonies, and all of the Leu^−^ colonies examined by sequence analysis were *leu2* mutants (Fig. 4), irrespective of the ratio levels. The guide sequences in the Cas9 plasmids mostly fit the strains (Table 2 (B), Fig. 4). However, the template DNA sequences differed by several nucleotides in some strains (Fig. 4). Therefore, the ratio could be increased by supplying a template DNA that perfectly matches the sequence around the guide sequence for each strain. We discuss an additional scenario due to the presence of the case where the template DNA sequence was not the issue of the template DNA sequence.

### Feasible reason for the cases of unexpectedly low Leu^−^ mutant ratio

In the DNA databases, no *LEU2* sequence was available for *S. kudriavzevii* CBS8840^T^, when we started this study. Sequence analysis of CBS8840^T^ strain’s *LEU2* revealed one nucleotide difference at 5’ end region of the target 20 bp compared with the common guide sequence for *Sc-SeLEU2* (Fig. 4E). The single-nucleotide difference at the site may have caused the low ratio, whereas one nucleotide substitution near the 3’-end of the 20 bp did not matter (Fig. 4A–D). The allodiploid strain *S. pastorianus* W34/70 harbors Sc-type and Se-type *LEU2* genes, similar to the strain CBS1513^T^ (Table 2 (B)). The guide sequence type Sc-SeLEU2 in the Cas9 plasmid was identical to the corresponding regions in the two *LEU2* genes in W34/70 (Fig. 4F) and CBS1513^T^ genomes (Table 2 (B)). One possible reason for the low Leu^−^ ratio is that an imaginary higher copy number of the genomic *LEU2* gene caused the lower Leu^−^ mutant ratio. In fact, de la Cerda Garcia-Caro et al. (2022) reported that the *S. pastorianus* W34/70 strain has multiple copies of *ScLEU2* and *SeLEU2*. It is feasible that the higher copy number of the *LEU2* gene in the genome caused the lower Leu^−^ mutant ratio.

In this study, we also predicted applicability of the *LEU2*-targeting plasmids and the template DNAs to other *Saccharomyces* sensu stricto species, namely, *S. cariocanus*, *S. mikatae*, *S. arboricolus*, *S. eubayanus*, and *S. uvarum*.

In conclusion, the series of CRISPR-Cas9 plasmids constructed in this study enabled the generation of *leu2* mutant strains from prototrophic strains of *Saccharomyces* sensu stricto species, including natural isolate, industrial, and allodiploid strains.

## Data Availability

Most of the plasmids and resultant leu2^−^ mutants produced in this study will be deposited in several repository organizations. The plasmid sequences also will be available there.
